# Mechanical and Electrical Properties of Free‐standing Polycrystal Diamond Membranes

**DOI:** 10.1002/advs.202503986

**Published:** 2025-06-28

**Authors:** Chenyu Wang, Dmitry Shinyavskiy, Luke Suter, Zubaida Altikriti, Quanxi Jia, Matthias Muehle, Jung‐Hun Seo

**Affiliations:** ^1^ Department of Materials Design and Innovation University at Buffalo The State University of New York Buffalo NY 14260; ^2^ Fraunhofer USA Inc Center Midwest East Lansing MI 48824 USA

**Keywords:** bandgap, chemical composition, diamond membranes, sheet resistance, youngs modulus

## Abstract

In this study, we demonstrate a novel approach for synthesizing free‐standing and transferable polycrystalline diamond membranes (PCDm) to overcome these constraints, thus enabling a much wider spectrum of applications. Two types of PCDm cantilevers —Top‐Surface‐Up (TSU) and Bottom‐Surface‐Up (BSU) are fabricated, each with two different sets of dimensions: 150 µm (width) × 1200 µm (length) and 300 µm (width) × 2000 µm (length). Their mechanical and electrical properties are systematically investigated. Atomic Force Microscopy (AFM) analysis revealed that TSU‐PCDm has a higher elastic modulus than BSU‐PCDm, attributed to differences in grain size and defect distribution. Despite these differences, all PCDms in our work exhibit consistently high modulus values with minimal mechanical degradation across various cantilever geometries. Bandgap measurements using X‐ray Photoelectron Spectroscopy (XPS) and UV–vis absorption spectroscopy indicated a lower bandgap for TSU‐PCDm due to boron incorporation, while BSU‐PCDm exhibited a higher bandgap due to increased hydrogen content. Electrical characterization showed that the sheet resistance of TSU‐PCDm decreases under strain, whereas BSU‐PCDm maintains stable resistance. These findings unveil the material properties of PCDm and their potential usage for myriad diamond‐based electronic applications.

## Graphical abstract

We demonstrate a novel approach for synthesizing free‐standing and transferable polycrystalline diamond membranes (PCDm). Two types of PCDm cantilevers—Top‐Surface‐Up (TSU) and Bottom‐Surface‐Up (BSU)—are fabricated and systematically analyzed for mechanical and electrical properties, including elastic modulus, Bandgap, sheet resistance under the bending conditions by tracing their changes in chemical compositions. We believe that this result unveils the material properties of PCDm and their potential usage for myriad diamond‐based electronic applications.

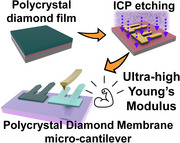

## 1 Introduction

Polycrystal diamond (PCD) exhibits many attractive material properties including excellent thermal properties such as thermal conductivity up to 1800 W K^−1^·m^−1^, which is close to that of single‐crystal diamond (SCD) (2200 W K^−1^·m^−1^),^[^ ^1^ ^]^ chemical inertness and stability at high temperatures,^[^ ^2^ ^]^ high hardness that can provide excellent wear resistance, superior tensile and fracture strength,^[^ ^3^ ^]^ and a wide range of tunability in conduction via doping or surface modification commonly with hydrogen and boron ions.^[^ ^4^, ^5^, ^6^ ^]^ Furthermore, unlike SCDs which suffer from a small substrate size, a PCD thin‐film can be grown on a variety of large‐area substrates up to a diameter of 300 mm with high film growth rates,^[^ ^7^ ^]^ excellent structural uniformity, and scalability, making it ideal for cost‐effective and large‐scale production.^[^ ^8^, ^9^ ^]^ Due to its unique material properties, PCD has been widely used for various cutting/grinding tools, surface protection layers, and heat sinks.^[^ ^10^, ^11^, ^12^ ^]^ For example, Yang et al, demonstrated a microchannel heat sink on a PCD film with a high thermal conductivity of 1500 W m^−1^·K^−1^ and achieved an effective cooling performance.^[^ ^13^ ^]^ Unique GaN‐on‐PCD heterostructures where the PCD thin film was directly deposited and wafer‐bonded were demonstrated to address the self‐heating effect of GaN devices.^[^ ^14^, ^15^ ^]^ Additionally, with advancements in micro‐ and nano‐fabrication technologies, PCD‐based advanced microelectromechanical systems (MEMS) structures that leverage the excellent mechanical, tribological, and chemical properties as well as the bio‐compatibility of PCD have been introduced for various bioelectronic applications.^[^ ^9^, ^16^, ^17^, ^18^, ^19^, ^20^, ^21^ ^]^

However, despite the superior material properties of PCD thin films, their device fabrication process relies on a top‐down approach due to the layered thin‐film geometry. Additionally, the available types of PCD‐based semiconductor devices are limited by the extremely rough top surface due to polycrystallinity. Polycrystalline materials, including PCD thin films, typically exhibit a mushroom‐like growth structure, where the bottom layer remains relatively smooth with small grains as it follows the surface roughness and texture of the handling substrate. However, as each grain grows upward, it enlarges, resulting in randomly oriented facets with roughness exceeding hundreds of nm.^[^ ^22^, ^23^ ^]^ This growth mechanism restricts the utilization of the PCD layer to the top surface, limiting access to the smoother bottom side. Consequently, this constraint hinders the broader application of PCD in a wider range of technologies.

In this study, a free‐standing and transferable format of the PCD layer, referred to as PCD membranes (PCDm hereafter), was developed to address the aforementioned challenges. Typically, semiconductor membranes refer to thin, standalone material forms that offer several key advantages, including a broad range of material availability and significant flexibility in manipulating shape, size, and thickness, while inheriting their bulk material properties.^[^ ^24^, ^25^ ^]^ As a result, they have recently been used in various applications where partially or fully fabricated semiconductor membrane‐based devices are required to be integrated onto inhomogeneous or lattice‐mismatched platforms.^[^ ^26^, ^27^, ^28^ ^]^ In particular, wide‐bandgap semiconductor membranes have recently been employed on wide‐ or ultrawide‐bandgap semiconductor platforms to realize high‐performance electronic and optoelectronic applications.^[^ ^29^, ^30^, ^31^ ^]^

Among various wide‐ and ultrawide‐bandgap semiconductors, diamond has received significant attention in recent years as a promising ultrawide‐bandgap material. However, the absence of a free‐standing and transferable form of PCD, namely PCDm, has hindered systematic investigation due to challenges associated with membrane synthesis and limited access to the bottom side of PCD thin films. Several approaches have been explored to address this challenge. Janssens et al. fabricated ultrathin nanocrystalline diamond membranes by growing a 150 nm boron‐doped film on glass, followed by backside HF etching through a patterned SU‐8 layer to release 555 µm diameter membranes for pressure sensing.^[^ ^32^ ^]^ Salvatori et al. produced diamond‐on‐silicon membranes via CVD growth and selective backside etching for similar applications.^[^ ^33^ ^]^ More recently, Jing et al. demonstrated a scalable exfoliation‐based method to generate ultrathin, ultraflat, and flexible diamond membranes directly from bulk substrates.^[^ ^34^ ^]^ While these approaches create PCD membranes and utilize the properties of PCD membranes, they are non‐transferable or bulk‐exfoliated approaches. In contrast, our method enables the pre‐patterning of PCDm into microcantilever geometries with controlled surface orientation, followed by release and transfer onto a foreign substrate. This surface‐selective transfer, combined with microfabrication compatibility, offers unique advantages for mechanical device integration and material property studies. The PCDm, which inherits excellent mechanical, electrical, and chemically stable properties from its bulk form, enables the realization of various electronic devices that leverage its outstanding material characteristics. For example, PCDm can serve as an active or conductive material for biocompatible sensors, as it can be easily reshaped using standard microfabrication processes while maintaining excellent chemical inertness and mechanical stability. Additionally, PCDm can be employed to develop cost‐effective, high‐performance power electronics or UV photodetectors by utilizing its favorable electrical and optoelectronic properties (namely, its wide bandgap and good charge transport characteristics) with great scalability. Given these material properties, PCDm not only enables the realization of the aforementioned high‐performance, cost‐effective, and scalable diamond electronics which can be compatible with or even superior to other wide‐ and ultra‐wide‐bandgap semiconductors, but also positions diamond as a major semiconductor material by facilitating its use as an active semiconductor or conductor with improved productivity and commercialization potential.

In this study, a simple synthesis process is demonstrated to separate PCDm from the bulk substrate, enabling the creation of high‐quality PCDm with excellent flexibility for manipulation into various shapes, the thus allowing for the investigation of its mechanical and electrical properties. To leverage the free‐standing nature of PCDm, two types of PCDm cantilevers — namely, Top‐Surface‐Up (TSU) and Bottom‐Surface‐Up (BSU) PCDm cantilevers — were fabricated and transferred onto the edge of a Si substrate, where they were permanently glued for mechanical property investigation, specifically the elastic modulus of PCDm, using Atomic Force Microscopy (AFM). Additionally, the electrical properties of PCDm, including bandgap and resistance under strain, were examined. X‐ray Photoelectron Spectroscopy (XPS) and Raman spectroscopy were conducted to further analyze the origin of differences in elastic modulus and bandgap between the surfaces of TSU‐ and BSU‐PCDm.

## 2 Experiment

**Figure 1:**
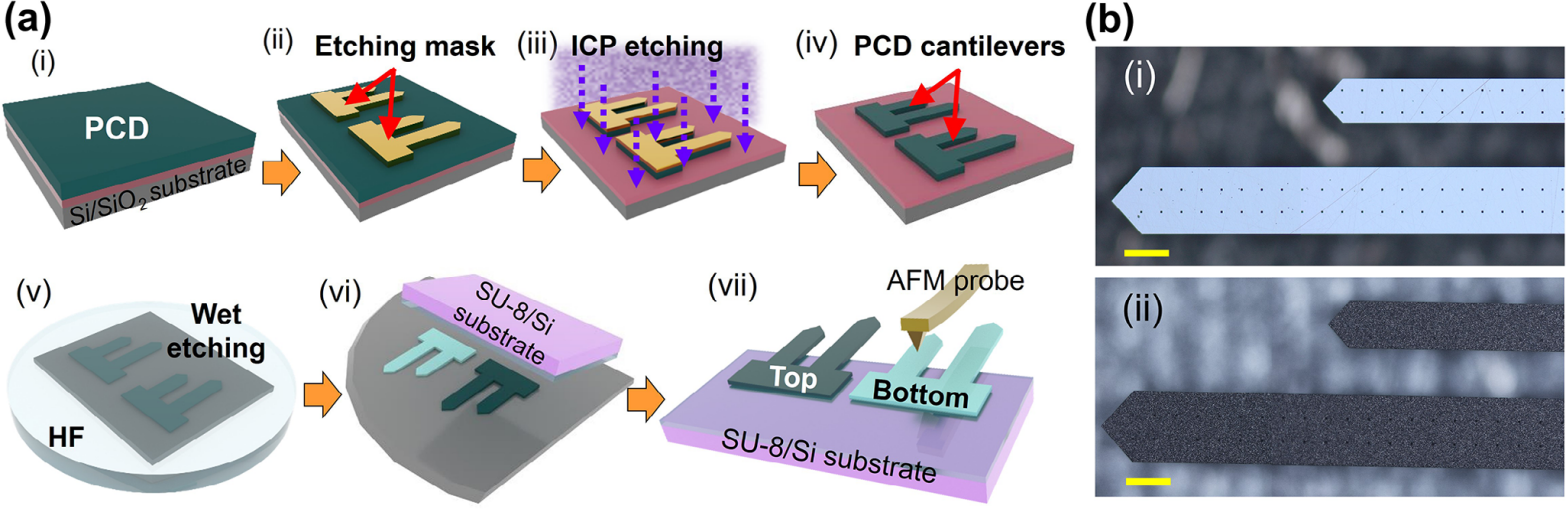
a) Schematic illustration of the fabrication process of the free‐standing PCDm cantilevers. b) Microscopic images of i) Bottom‐Surface‐Up (BSU) and ii) Top‐Surface‐Up (TSU) PCDm cantilevers (the scale bar is 200 µm).

**Figure** **1**a shows the schematic illustration of the preparation process of PCDm and PCDm‐based cantilevers. The details of the fabrication processes can be found in the Method section. In brief, the PCDm cantilever fabrication begins with the growth of poly‐crystalline diamond films on (100) Si/SiO_2_ wafers by microwave plasma‐enhanced chemical vapor deposition (MPECVD) technique, as shown in Figure 1a‐i. Two different types of cantilever patterns that have different lengths were formed using a photolithography process (i.e., the width of the long cantilever is 300 µm and that of the short cantilever is 150 µm.) (Figure 1a‐ii), followed by an inductively coupled plasma ‐ reactive ion (ICP‐RIE) etching using a Cr/Ni bi‐layered metal etching mask.^[^ ^35^ ^]^ (Figure 1a‐iii,iv) Then, the entire sample was immersed in a hydrofluoric (HF) acid solution to remove the underlying SiO_2_ layer, which separates the cantilever‐shaped PCD layer from the Si/SiO_2_ substrate. The released cantilever‐shaped PCD layer, now referred to as PCDm, was carefully transferred by a micro‐transfer printing technique^[^ ^36^, ^37^, ^38^ ^]^ at the edge of the adhesive (SU‐8) coated Si substrate to form free‐standing PCDm cantilevers. (Figure 1a‐v–vii) During the transfer step, two additional varieties of PCDm cantilevers were created: the Top‐Surface‐Up (TSU) and the Bottom‐Surface‐Up (BSU) PCDm cantilevers, due to the flexibility of the transfer process to manipulate the PCDm.^[^ ^24^ ^]^ It is important to note that this variety, which enables access to the bottom surface of the PCDm, otherwise not accessible in the thin‐film format, allows for a direct comparison of the mechanical and electrical properties of TSU‐ and BSU‐ PCDm. As discussed in the following section, this difference originates from the mushroom‐like vertical structure of the PCD grains, which leads to significant differences in the chemical composition, grain size, and dopant concentrations between the top and bottom sides of the PCD layer.

## 3 Results and Discussion

**Figure 2:**
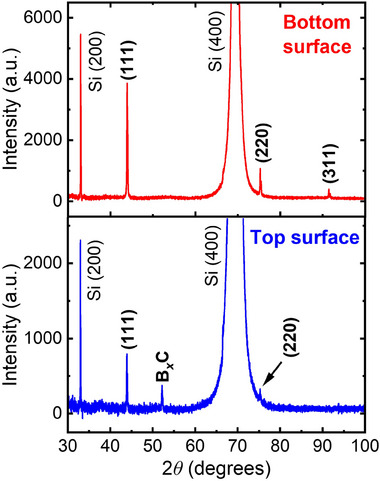
A comparison of X‐ray diffraction patterns of BSU‐ and TSU‐ PCDm cantilevers.

To investigate the structural properties and phase composition of the polycrystalline diamond membranes (PCDms), we performed X‐ray diffraction (XRD) measurements on both the top and bottom surfaces. **Figure** **2** shows the corresponding diffraction patterns of the TSU‐ and BSU‐PCDms. In addition to the substrate (Si) peaks at 2*θ* ≈ 33° and 69°,^[^ ^39^ ^]^ both surfaces exhibit sharp and well‐defined diamond peaks at 2*θ* ≈ 43.7° and 75.2°, corresponding to the (111) and (220) planes of diamond, respectively, confirming high crystallinity. A peak at 2*θ* ≈ 91.3°, corresponding to the (311) plane of diamond, is observed only on the bottom surface—likely due to its higher overall diffraction intensity, which results from a smoother surface morphology. Notably, the peak intensity ratio between the (111) and (220) reflections is ≈4:1 for both surfaces, which is characteristic of randomly oriented diamond grains.^[^ ^40^ ^]^ This suggests that the diamond grains on both surfaces are randomly oriented, with no evidence of strong crystallographic texture. Furthermore, the peak positions are identical between the two surfaces, indicating minimal residual strain. The full width at half maximum (FWHM) of the (111) peak is slightly broader for the bottom surface than for the top surface, which may reflect a smaller average coherent domain size or greater microstructural disorder. Additionally, a distinct diffraction peak at 2θ ≈ 52° appears exclusively on the top surface, which is attributed to the formation of a boron‐carbon (B*_x_* C) phase as a result of boron doping during growth.^[^ ^41^ ^]^

**Figure 3:**
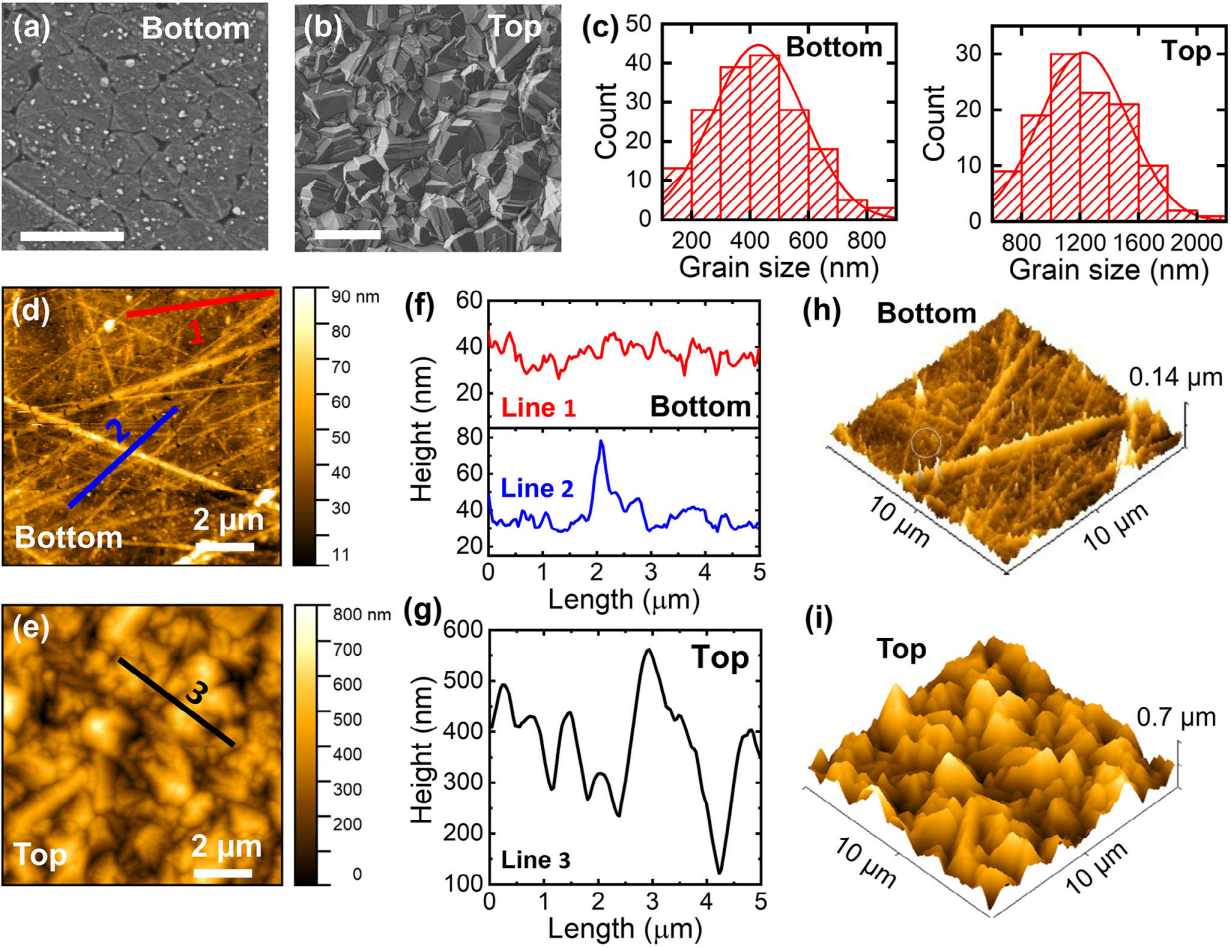
(a, b) SEM morphologies (the scale bar is 1 µm) and (c, d) the statistical distribution of the grain size of TSU‐ and BSU‐ PCDm. (d, e) AFM topographical images, (f, g) corresponding line profiles, and (h, i) 3D images of BSU‐ and TSU‐ PCDm, respectively.

**Figure 3**a,b compares the surface morphology of the BSU‐ and TSU‐ PCDm cantilevers. Both surfaces display distinct polycrystalline grain boundaries; however, the surface of the BSU‐PCDm is significantly smoother than that of the TSU‐PCDm, because the BSU‐PCDm surface replicates the surface roughness of the Si substrate during the growth process. As shown in Figure 3c, the statistical distribution of the grain size on both surfaces was examined using a visual analysis technique with multiple SEM images, resulting in average grain sizes of ≈1200 and 500 nm for the top and bottom surfaces of the PCDm, respectively, which accounts for the mushroom‐like vertical structure of the PCD grains. To further understand the topographical features of both types of PCDm, atomic force microscopy (AFM) was performed to investigate their atomic‐scale surface roughness. As shown in Figure 3d,e the topographical images were obtained on the surface of BSU‐ and TSU‐ PCDm, respectively. The height range, with distinct differences of ≈80 and 800 nm on the bottom and top surfaces of the PCDm, confirms that the roughness of the top surface is ≈10 times greater than that of the bottom surface. Figure 3f,g which presents height profiles from AFM images also confirms that the roughness of the bottom and top PCDm surfaces is noticeably different. This difference in surface roughness can be clearly seen in three‐dimensional AFM images in Figure 3h,i as well. It should be noted that several scratch‐like lines crossing the surface of the BSU‐PCDm, as shown in Figure 3d, were created during the mechanical polishing of the Si substrate with diamond slurry. This surface preparation step was conducted prior to diamond growth to promote initial carbon nucleation at the Si/diamond interface. Since the mechanical properties of polycrystalline materials are significantly influenced by grain size,^[^ ^42^ ^]^ the two differently surfaced PCDm, namely TSU‐ and BSU‐ PCDm, are expected to exhibit different stiffnesses due to differences in grain size and packing density as shown in Figure 3. Therefore, it is essential to investigate the spring constant and Young's modulus of these PCDm, which govern their stiffness. To investigate the mechanical properties of micro‐scale TSU‐ and BSU‐ PCDm cantilevers, force plots were obtained using AFM. An AFM probe with a diamond‐coated tip and a high spring constant was employed to apply sufficient force to bend the PCDm cantilevers. (The key parameters of the AFM probe are provided in Table S1).

The actual spring constant of the AFM probe, *k*_AFM_ (111.35 N/m), was calibrated before the measurement and used for calculation for the spring constant and Young's modulus of the TSU‐ and BSU‐PCDm cantilevers. The scheme that illustrates the probing points for the bending test can be found in Figure S2. The length values (*L*) represent the distance of the probing spot on the PCDm cantilever from the edge of the Si substrate, where the load is applied. The probe tip was positioned along the centerline of the PCDm cantilever, and measurements were conducted at intervals of 100 µm from the edge of the Si substrate toward the end of the PCDm cantilever. The trigger point of displacement is set to 70 nm for each approach and the force plot (‘Force’ versus ‘*Z*‐axis displacement’) of each spot was recorded. Also, the lateral voltage was monitored and only the force plot with a lateral voltage change within 40 mV was used to avoid unwanted interference of lateral force. Force plots of each probing spot (*L*) were measured as shown in Figure S3 and the effective stiffness (*k*_eff_) which represents the spring constant of the PCD cantilever and the AFM cantilever were calculated from the linear slope shown in Figure S3.

To extract the spring constant of the PCDm cantilevers, Equation 1 was used, where *k*_AFM_ and *k*_PCD_ are the spring constant of the AFM probe and the PCDm cantilevers, respectively.^[^ ^43^, ^44^ ^]^

(1) $$k_{\mathrm{eff}}=\frac{1}{\frac{1}{k_{\mathrm{AFM}}}+\frac{1}{k_{\mathrm{PCD}}}}$$

Then, Equation 2 was used to fit the *k*_PCD_ vs *L* relationship, where *E* is the elastic modulus of PCDm cantilevers, *I* is the areal moment of inertia of the cantilever's cross‐section, 𝑣 is Poisson's ratio (a value of 0.0718 for polycrystal diamond is used from Ref 45.^[^ ^45^ ^]^), and *L* is the distance from the edge of the Si substrate to the probing point, and *L*_0_ is a correction length for *L*.^[^ ^46^ ^]^

(2) $$k_{\mathrm{PCD}}=\frac{3EI}{\left(1-v^{2}\right){\left(L+L_{0}\right)}^{3}}$$

Since the cross‐section of the PCDm cantilevers is rectangular, the areal moment of inertia is calculated using the Equation 3,^[^ ^47^ ^]^ where *w* is the width, and *t* is the thickness of the PCDm cantilever (*t* = 3.8 µm). In addition, the *L*_0_ values for all samples are nearly two orders of magnitudes smaller than *L* which indicates that the *L* value is a dominant factor in this calculation.

(3) $$I=\frac{wt^{3}}{12}$$

**Figure 4:**
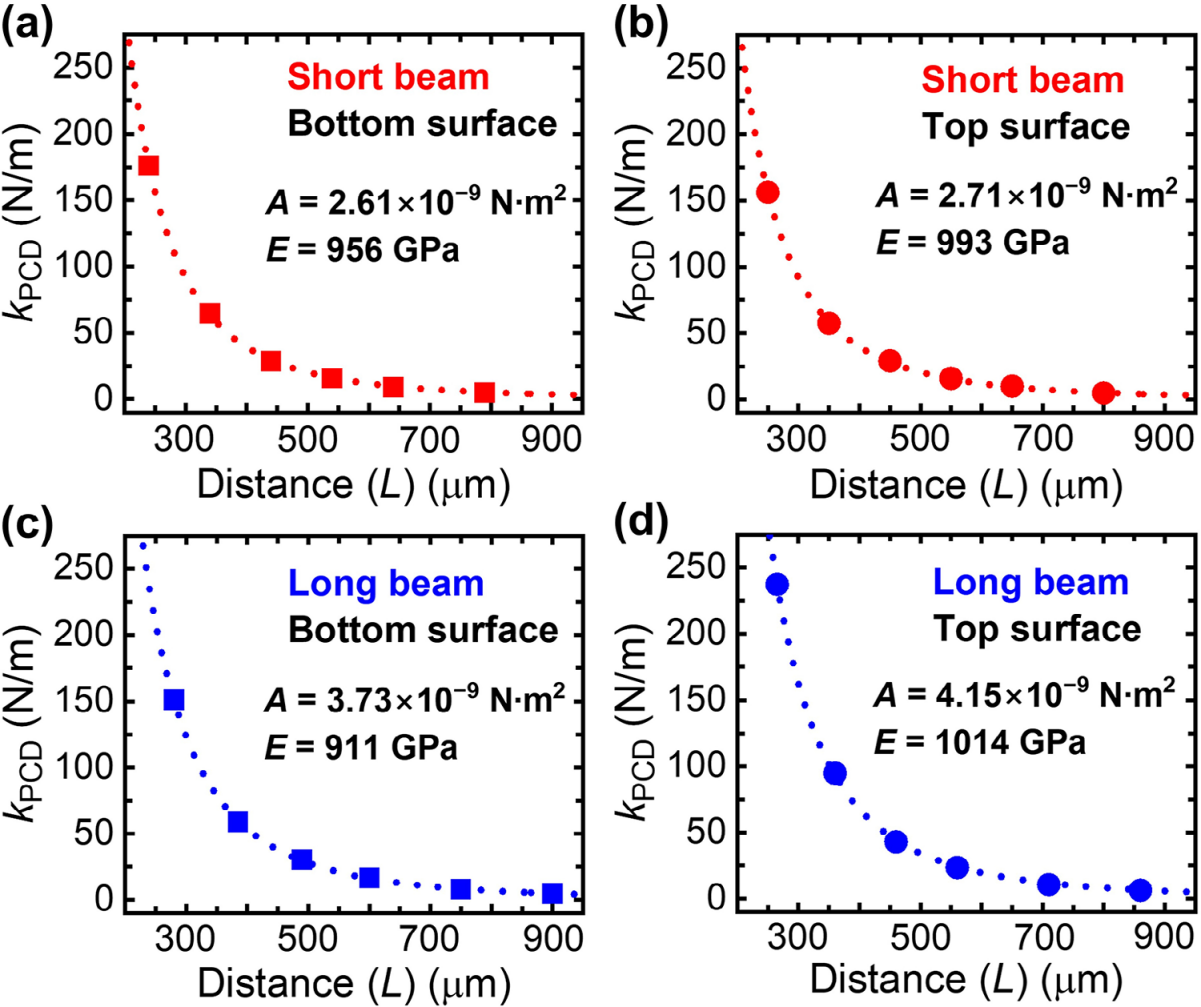
Measured and fitted plot of *k*_PCD_ as a function of the distance (*L*) of a) the short BSU‐PCDm cantilever, b) the short TSU‐PCDm cantilever, c) the long BSU‐PCDm cantilever, and d) the long TSU‐PCDm cantilever.

As shown in **Figure** **4**a–d, experimental data points fit well to the calculated *k*_PCD_ *vs. L* fitted line using Equation 2 with the parameter *A* = [3·*I* / (1 − 𝑣^2^)] × *E*. Since short and long PCDm cantilevers have different cross‐sectional areas, the constant, 3·*I* / (1 − 𝑣^2^), is equal to 2.73 × 10^−21^ and 4.09 × 10^−21^ m^4^ for short and long PCDm cantilevers, respectively. The short BSU‐PCDm cantilever exhibits an elastic modulus (*E*) of 956 GPa, while the short TSU‐PCDm cantilever shows an *E* value of 993 GPa. For the long cantilevers, the elastic modulus is 911 GPa for the BSU‐PCDm and 1014 GPa for the TSU‐PCDm.

**Figure 5:**
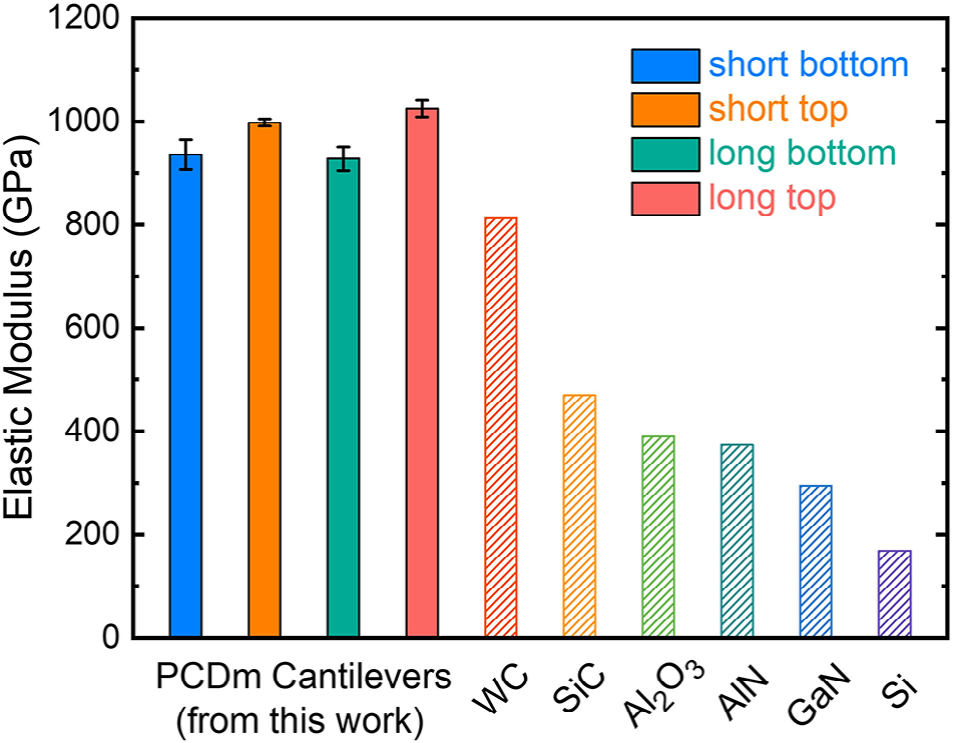
Elastic modulus of PCDm cantilevers compared with other wide bandgap semiconductors and ceramics.

Interestingly, the elastic modulus values of the TSU‐ and BSU‐ PCDm cantilevers remain constant regardless of their lengths, suggesting that the elastic modulus of the PCDm cantilevers is not significantly affected by variations in width or length. Furthermore, both the long and short cantilevers with the TSU‐PCDm exhibit slightly higher elastic modulus values compared to those with the BSU‐PCDm. Commonly, for polycrystal materials, hardness increases as grain size decreases due to the strengthening effect of grain boudaries according to Hall‐Petch relationThis difference can be attributed to the Hall‐Petch relation, which describes an increase in hardness as grain size decreases due to the strengthening effect of grain boundaries in polycrystalline materials.^[^ ^48^ ^]^ The overall mechanical stiffness increases as the average grain size decreases as more grain boundaries act as obstacles to dislocation movement. However, in this case, the BSU‐PCDm cantilever, which has a smaller average grain size, exhibits a lower elastic modulus compared to the TSU‐PCDm cantilever with a larger average grain size. Therefore, we speculate that other processing factors,^[^ ^49^ ^]^ such as impurities in the PCDm or growth rate, may influence the mechanical properties of the PCDm. **Figure** **5** compares the elastic modulus of all four types of PCDm cantilevers from this work with other materials including Si,^[^ ^50^ ^]^ wide bandgap semiconductors (SiC^[^ ^51^ ^]^ and GaN^[^ ^52^ ^]^), ceramics (Al_2_O_3_^[^ ^53^ ^]^ and AlN^[^ ^54^ ^]^), and cermet (Tungsten carbide (WC)^[^ ^55^ ^]^). Surprisingly, the elastic modulus of PCDm cantilevers is more than twice as high as that of other wide‐bandgap semiconductors and ceramics, including SiC, which is well‐known for its excellent mechanical properties. Only WC exhibits a slightly lower elastic modulus than the PCDm cantilevers. However, its lack of corrosion resistance due to the metallic binder presents a clear disadvantage compared to the PCDm.^[^ ^56^ ^]^ Compared to single‐crystal diamond, which has the highest experimental elastic modulus of ≈1130 GPa,^[^ ^57^ ^]^ the PCDm cantilevers fabricated in this work approach this upper limit while offering significantly greater design flexibility. Single‐crystal diamond is typically constrained to small and fixed geometries due to growth limitations and processing challenges. In contrast, our free‐standing and transferable PCDm can be readily fabricated in customized dimensions and integrated into various device architectures. Furthermore, previously reported polycrystalline diamond films generally exhibit a wide range of elastic modulus values, depending on factors such as grain size, porosity, and internal stress levels. For instance, Klein et al. reported an elastic modulus of ≈1143 GPa for high‐quality polycrystalline diamond films,^[^ ^45^ ^]^ while other studies have observed values as low as 500 GPa due to microstructural imperfections.^[^ ^58^ ^]^ Such variability often leads to mechanical instability or compromised device performance. In contrast, PCDms in our work demonstrate consistently high modulus values with minimal mechanical degradation across different cantilever geometries. Overall, the free‐standing and transferrable PCDm cantilever demonstrates a superior elastic modulus, with its mechanical properties exhibiting minimal variation across different dimensions, thereby offering greater flexibility in tailoring them to desired sizes and shapes in various electronic applications.

**Figure 6:**
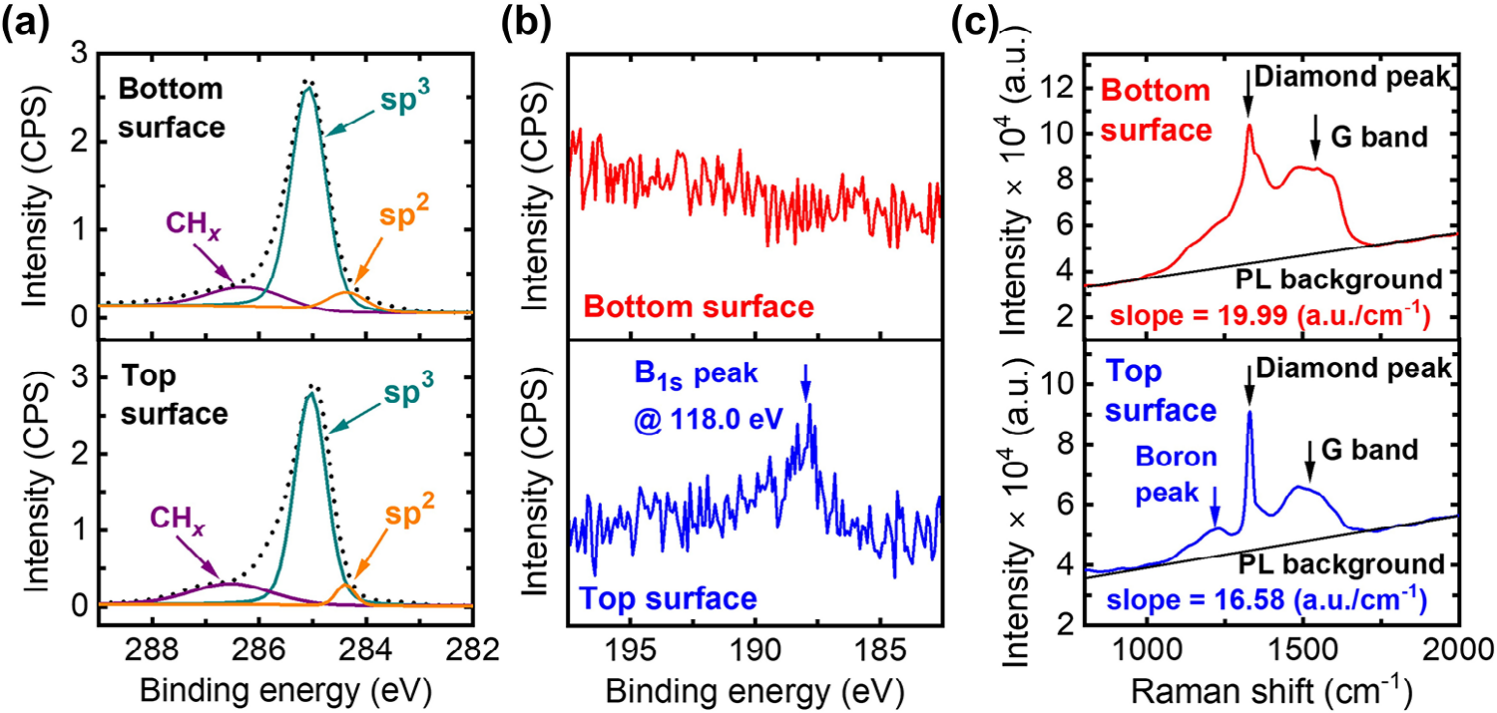
a) Deconvolution of the XPS carbon 1s high‐resolution spectra, b) XPS boron 1s spectra, and, c) Raman spectra of the BSU‐ and TSU‐ PCDm.

X‐ray photoelectron spectroscopy (XPS) and Raman measurements were employed to analyze the chemical composition of the surfaces of the TSU‐ and BSU‐ PCDm. As shown in **Figure** **6**a, The XPS C1s core‐level spectra are centered at ≈285 eV for both surfaces which were deconvoluted into three components centered at 286.5, 284.4, and 285.0eV to further analyze hydrogen–carbon (CH*_x_*), sp^2^, and sp^3^ species.^[^ ^59^ ^]^ The peaks at 284.4 and 286.5eV are attributed to sp^2^ carbon (C═C) species and CH*_x_* species, respectively. The areas covered by these deconvoluted peaks were used to calculate the ratio of these two types of impurities: sp^2^ content and hydrogen. (Calculated values can be found in Table S2.) The sp^2^/sp^3^ ratios of 0.104 and 0.059 and the CH*_x_*/sp^3^ ratios of 0.263 and 0.237 were calculated from the BSU‐ and the TSU‐ PCDm surfaces, respectively. In addition, as shown in the XPS high‐resolution spectra in Figure 6b, the boron 1s peak at 188.0 eV appears only on the top PCDm surface, indicating that boron is predominantly incorporated near the top surface. (The XPS survey scan is provided in Figure S4.)

Since the XPS technique is surface‐sensitive, Raman spectroscopy, which probes deeper into the PCDm, was employed to further validate the XPS results. In the Raman spectrum, sp^3^ carbon and sp^2^ carbon exhibit distinct differences, enabling the assessment of the sp^2^ ratio. Furthermore, it has been reported that the hydrogen concentration is proportional to the slope of the photoluminescence background,^[^ ^60^ ^]^ therefore the hydrogen ratio can also be analyzed using Raman spectroscopy. As shown in Figure 6c, the diamond (sp^3^) peaks located at 1332 cm^−1^ are the most intensive peaks for both TSU‐ and BSU‐ PCDm. It corresponds well with the sp^3^ peaks in the XPS results, indicating that the sp^3^ content dominates both the bottom and top surfaces of the PCDm. The broadened peak at ≈1550 cm^−1^ can be assigned to the G (sp^2^) band. The sp^2^ / sp^3^ ratio is calculated using the following formula, *f* = 100 × *I*_G_ /(75 × *I*_diam_ + *I*_G_)^[^ ^61^, ^62^ ^]^ yielded to be 0.008 for the surface of the BSU‐ PCDm and 0.004 for the surface of the TSU‐ PCDm, respectively. In addition to intensity ratios, the full width at half maximum (FWHM) of the diamond peak provides insight into structural quality. The FWHM of the TS‐PCDm is narrow without any peak shifting (≈20 cm⁻¹), indicating high crystallinity and uniform stress distribution. In contrast, the BS‐PCDm exhibits a significantly broader FWHM of ≈60 cm⁻¹, suggesting greater disorder. Moreover, the diamond peak of the BS‐PCDm shows a clear asymmetry with a shoulder toward higher wavenumbers, which is attributed to a weak D band of sp^2^ carbon (≈1380 cm⁻¹) after deconvolution.^[^ ^63^ ^]^ The slopes of the PL background for the bottom and top surfaces of the PCDm were also calculated to evaluate hydrogen content.^[^ ^57^ ^]^ The PL slope of the BSU‐PCDm was found to be 19.99 (a.u./cm^−1^) whereas that of the TSU‐ PCDm was 16.58 (a.u./cm^−1^). Notably, a weak boron‐induced lattice distortion peak at ≈1216 cm^−1[^ ^63^ ^]^ is observed exclusively on the TS‐PCDm, consistent with the findings from the XPS analysis. As summarized in Table S2, both XPS and Raman analyses indicate higher sp^2^ and hydrogen content on the surface of the BSU‐PCDm compared to the TSU‐PCDm, which aligns well with the results by others.^[^ ^59^ ^]^ XPS and Raman spectroscopy indicate that the bottom surface of PCDm has a higher sp^2^ and hydrogen content than the top surface. The asymmetric distribution of defects between the top and the bottom of the PCD can be explained by the uneven defects incorporation into the grain boundaries^[^ ^49^, ^64^ ^]^ thus leading to a decrease in elastic modulus. On the other hand, according to the Hall‐Petch relationship,^[^ ^48^ ^]^ denser grains with more grain boundaries act as obstacles to dislocation movement, leading to an enhancement of the elastic modulus. In PCDm, the bottom surface has a smaller grain size (<500 nm) compared to the top surface (>1200 nm). Consequently, despite its higher defect content, the bottom surface exhibits only a slightly lower elastic modulus than the top surface for both long and short beams.

**Figure 7:**
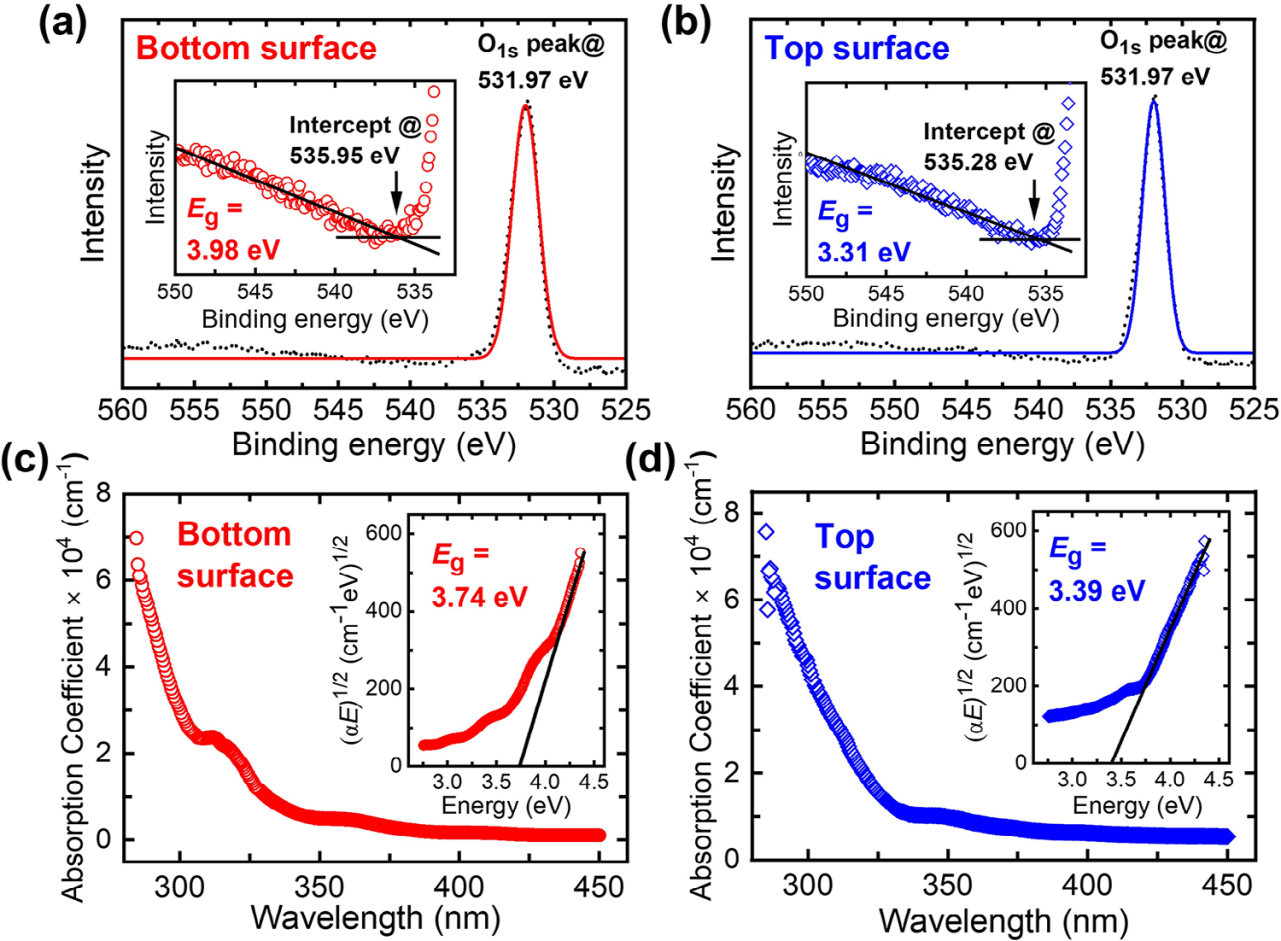
a,b) XPS high‐resolution spectra of oxygen 1s peak and c,d) absorption spectra and (Insets) Tauc plots of BSU‐ and TSU‐ PCDm.

In addition to its excellent mechanical properties, bandgap characterization is essential to assess the potential of PCDm as a candidate for wide bandgap semiconductors. The bandgap of PCDm was determined using XPS and UV–vis absorption spectroscopy. As shown in **Figure** **7**a,b, the plasma loss peak was observed for both the surface of the BSU‐ and the TSU‐ PCDm at ≈555 eV next to the O 1s core level peak with a fitted value of 531.97 eV. The bandgap of these samples were measured to be 3.98 eV for the BSU‐ PCDm, and 3.31 eV for the TSU‐PCDm, respectively, as shown in the insets of Figure 7a,b.^[^ ^65^ ^]^ To validate the bandgap of BSU‐ and the TSU‐ PCDm, the UV–vis absorption spectroscopy was also used, as shown in Figure 7c,d. According to the absorption function:

(4) $$\alpha h\nu =C{\left(h\nu -E_{g}\right)}^{1/2}$$

where *α* is the absorption coefficient, h𝑣 is the photon energy *E*, and C is a constant, the bandgap value can be calculated by extrapolating the linear portion of the Tauc plot to the energy axis as shown in Figure 7c,d.^[^ ^31^ ^]^ The bandgap of the BSU‐PCDm is 3.74 eV, while that of the TSU‐PCDm is 3.39 eV. This difference in the bandgap values can be attributed to the variation in chemical composition difference between the top and bottom surface in the PCDm. For example, it has been reported that the three main types of impurities ‐ boron, sp^2^, and hydrogen – significantly influence the bandgap value of PCDm. i) An increase of boron concentration results in a decrease in the optical bandgap or a shift of the absorption peak to a lower wavenumber,^[^ ^66^, ^67^ ^]^ ii) A higher hydrogen content leads to an increase in the bandgap,^[^ ^68^ ^]^ iii) The bandgap value decreases linearly as the sp^2^/sp^3^ ratio increases.^[^ ^69^, ^70^ ^]^ Previous analysis suggests that the bottom surface of PCDm contains slightly higher sp^2^ and hydrogen contents, whereas boron is primarily incorporated on the top surface of PCDm. Consequently, the lower bandgap value on the TSU‐PCDm can be attributed to boron incorporation and the relatively lower hydrogen content compared to BSU‐PCDm. On the other hand, the sp^2^/sp^3^ ratio on the bottom surface is only slightly higher than that of the top surface, with a value less than 0.05, indicating that its influence on the overall bandgap difference is minimal.

**Figure 8:**
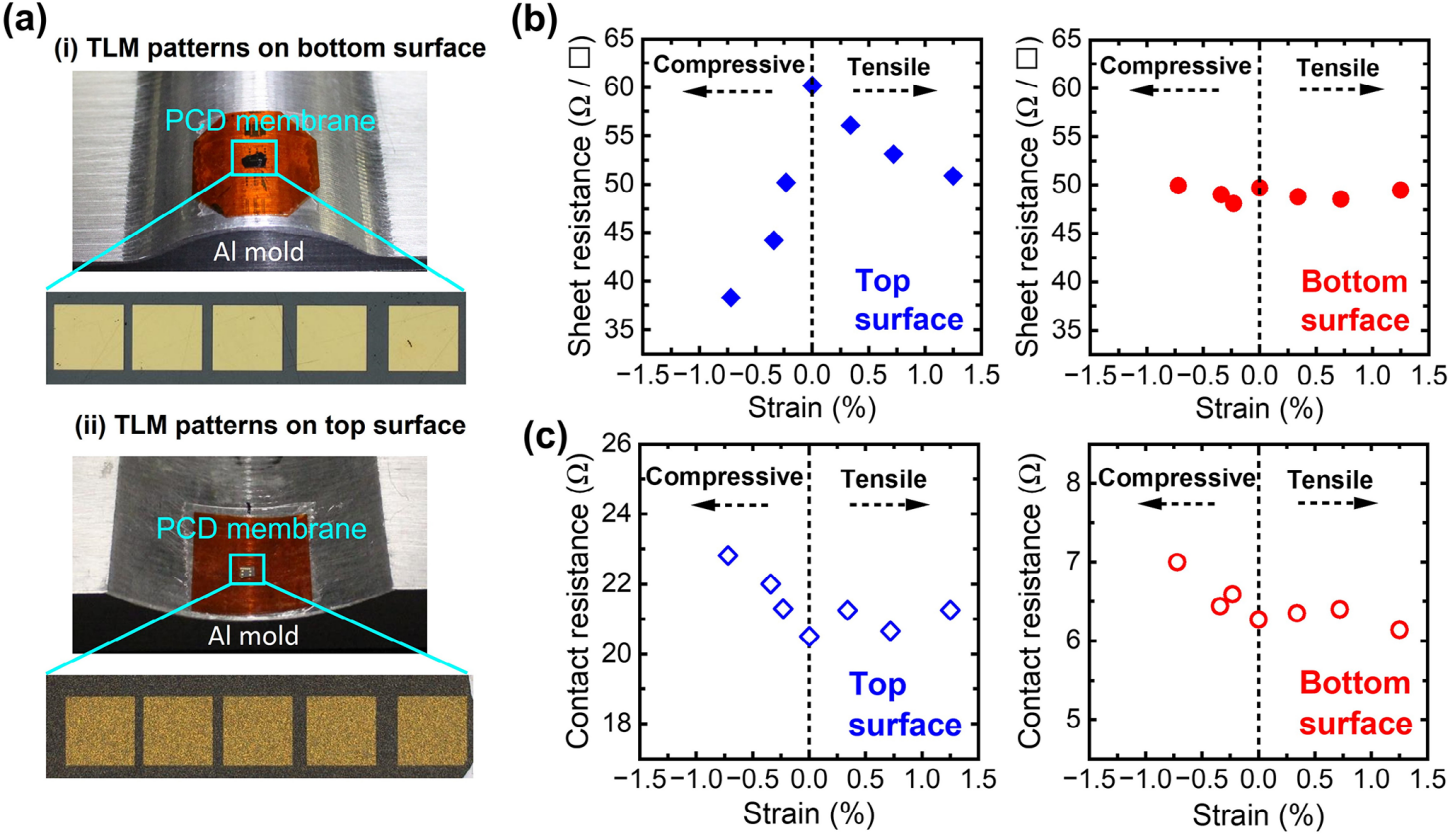
a) Microscopic images of TLM patterns on PCDm under bending conditions. b,c) Sheet resistance and contact resistance of top and bottom surface of PCDm under different strain conditions.

**Figure 9:**
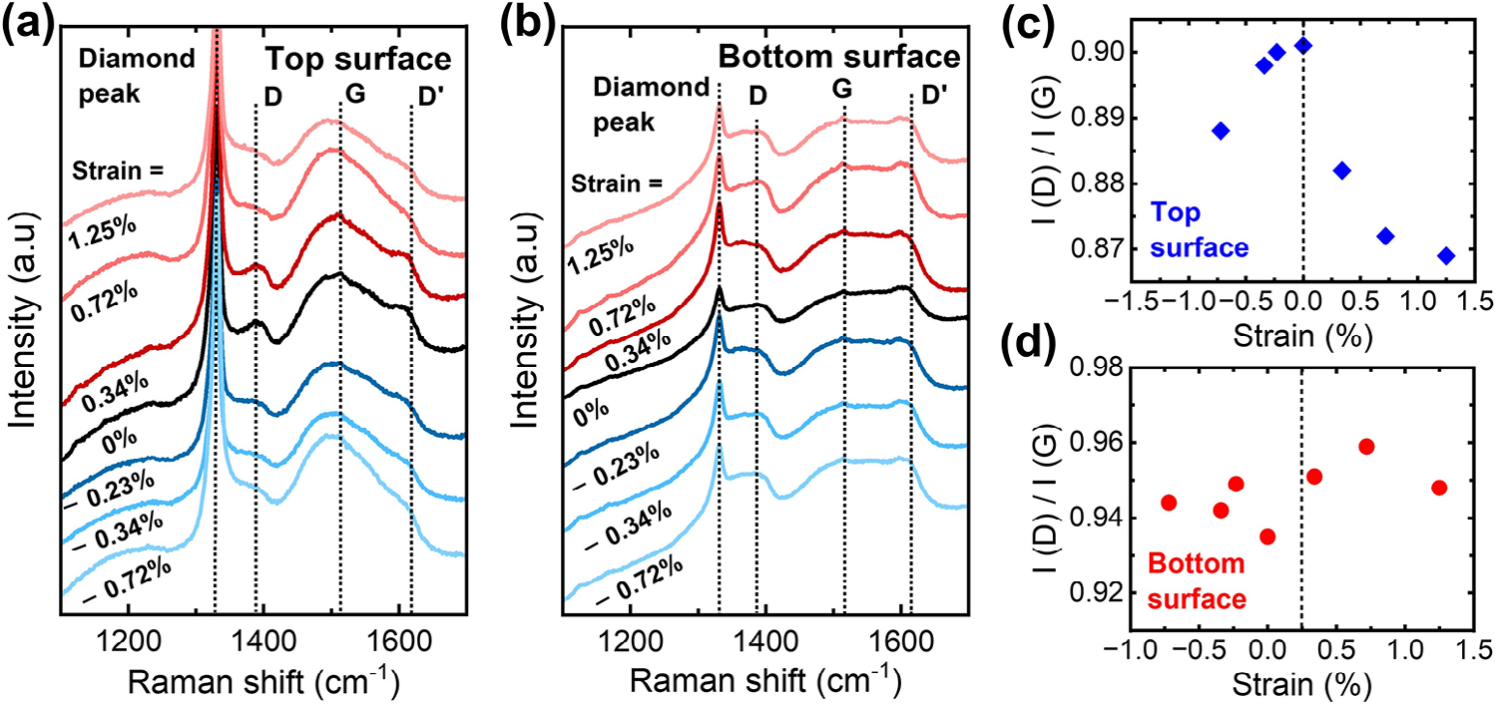
a,b) Raman spectra and c,d) The ratio of D band and G band intensity of TSU‐ and BSU‐ PCDm under different strain conditions.

Similar to the variations in bandgap observed in TSU‐ and BSU‐ PCDm, differences in material properties, such as impurity content and grain size, can directly affect their electrical properties. To investigate changes in the resistivity of PCDm, the transmission line method (TLM) was employed. Furthermore, the free‐standing format of PCDm enables a more systematic investigation of its electrical properties, including various bending tests under biasing conditions. As shown in **Figure** **8**a, the TLM pattern was fabricated on the PCDm prior to the transfer step and glued onto a polyimide substrate with SU‐8 as an adhesive. Convex and concave aluminum molds with different curvatures were employed to apply various strain conditions to the PCDm, ranging from −0.8% compressive strain to 1.3% tensile strain. The sheet resistance and contact resistance of the PCDm were determined by analyzing the *I–V* curves. The total resistance (*R*_T_) change with length (*L*) under different strains can be found in Figures S5 and S6. Figure 7b presents the variations in the sheet resistance (*R*_s_) of TSU‐ and BSU‐ PCDm under different strain conditions. Under the flat condition, the sheet resistance of the TSU‐PCDm (60.19 Ω sq^−1^) is higher than that of the BSU‐PCDm (49.7 Ω sq^−1^). This difference in the sheet resistance can be attributed to the sp^2^/sp^3^ ratio, as a higher sp^2^ content leads to more conductive current paths across the grains. Interestingly, the sheet resistance of TSU‐PCDm decreases with strain, showing a more noticeable reduction under compressive strain. In contrast, the sheet resistance of BSU‐PCDm remains nearly unchanged under strain. As shown in Figure 8c, the contact resistance of TSU‐PCDm is significantly higher (20.50 Ω sq^−1^) than that of BSU‐PCDm (6.27 Ω sq^−1^), and the contact resistance for both surfaces is unaffected by strain. To further investigate the difference in the strain‐dependent sheet resistance of TSU‐ and BSU‐PCDm surfaces, Raman spectroscopy was performed under strain conditions. As shown in **Figure** **9**a,b, the diamond peak at 1332 cm^−1^ is attributed to sp^3^ carbon, while the other characteristic peaks (D, G, and D′ bands) are associated with sp^2^ carbon. The G band, located at ≈1510 cm^−1^, reflects the in‐plane bond stretching motion of sp^2^ carbon, while the D band, at ≈1380 cm^−1^, indicates structural disorders in the graphitic structure that arise from out‐of‐plane vibration. With increasing disorder, the D band develops a shoulder, referred to as the D′ band, at ≈1620 cm^−1^.^[^ ^71^, ^72^, ^73^, ^74^ ^]^ The D and D′ bands are most prominent on the surface of TSU‐PCDm under flat conditions but gradually diminish as strain increases. This trend suggests that the disorder level of sp^2^ carbon decreases with strain, resulting in a reduction in sheet resistance. However, the relative intensity of the D and D′ bands on BSU‐PCDm remains unchanged with strain, indicating a more stable sheet resistance of the bottom surface. The ratio of the intensity of D and G peaks was calculated and shown in Figure 9c,d. For the surface of the TSU‐PCDm, the *I*(*D)*/*I*(*G*) ratio is the highest under flat conditions and decreases with increasing strain. This further confirmed the dominance of the D band which indicates the highest disorder level when no strain is applied. In contrast, for the surface of the BSU‐PCDm, the *I*(*D)*/*I*(*G*) ratio remains relatively constant with increasing strain, consistent with the observation that the sheet resistance of the bottom surface of PCDm remains stable.

## 4 Conclusion

This study introduces a novel approach for fabricating free‐standing and transferable PCDm to address the challenges associated with conventional PCD thin films. The PCDm were synthesized using a simple separation process, allowing for enhanced mechanical flexibility and access to both top and bottom surfaces. Two types of PCDm cantilevers—Top‐Surface‐Up (TSU) and Bottom‐Surface‐Up (BSU)—were fabricated to analyze their mechanical and electrical properties. The mechanical properties were investigated using AFM, revealing that the TSU‐PCDm exhibits a higher elastic modulus than the BSU‐PCDm, attributed to differences in grain size and defect incorporation. Additionally, bandgap measurements through XPS and UV–vis absorption spectroscopy demonstrated that the BSU‐PCDm has a higher bandgap due to increased hydrogen content and reduced boron incorporation. Electrical characterization showed that TSU‐PCDm has a higher sheet resistance, which decreases under strain due to structural disorder variations in sp^2^ carbon content. In contrast, BSU‐PCDm maintains a stable sheet resistance under strain. The work we demonstrate here paves the way for expanding the use of PCD in electronic applications as a promising wide‐bandgap semiconductor candidate. For instance, PCDm can be used as an active or conductive material in biocompatible sensors, benefiting from its ability to be easily reshaped through standard microfabrication processes while retaining excellent chemical inertness and mechanical stability. Moreover, its wide bandgap, excellent charge transport characteristics, and broad doping concentration range enable the development of cost‐effective, high‐performance power electronics and UV photodetectors with excellent scalability. Owing to these material advantages, PCDm is not only a strong candidate for high‐performance, scalable, and economically viable diamond electronics—potentially outperforming other wide‐ and ultra‐wide‐bandgap semiconductors—but also strengthens diamond's position as a major semiconductor material by enhancing its applicability as an active semiconductor or conductor with improved productivity and commercialization prospects.

## 5 Experimental Section

### PCD Cantilever Fabrication

Figure 1a illustrates the schematic preparation process of PCDm cantilevers. The boron‐doped diamond film was grown using microwave plasma‐enhanced chemical vapor deposition (MPECVD) under a pressure of 60 Torr with a forward power of 7 kW. The gas mixture consisted of 8 sccm CH₄, 385 sccm H₂, and 7.5 sccm B₂H₆. The substrate underwent scratch seeding for 5 min with 0–2 µm diamond powder to enhance nucleation density. The deposition process lasted 6 h and 45 min, reaching a temperature of 936°C at 45 min. The resulting diamond film exhibited a thickness of 3.8 µm, with a sheet resistance of 74.8 Ω and a calculated resistivity of 2.71 × 10⁻^2^ Ω·cm. The thickness of the PCD thin film was measured to be 3.8 µm using a surface profilometer (Profilm 3D). (Figure 1a‐i) As shown in Figure 1a‐ii, a metal stack consisting of Cr (100 nm) and Ni (300 nm) was deposited on the PCD surface using an electron‐beam evaporator (Kurt J. Lesker Company AXXIS) to serve as an etching mask. Inductively coupled plasma–reactive ion etching (ICP‐RIE) (Trion Technology Oracle III) was then employed to selectively remove the unmasked portions of the PCD layer, exposing the underlying SiO₂ layer (Figure 1a‐iii,iv). At this stage, the separated PCD layer is referred to as PCDm. The remaining metal mask was subsequently removed using a metal etchant (ceric ammonium nitrate and nitric acid, 6%), followed by immersion in hydrofluoric acid (HF, 49%) to dissolve the SiO₂ layer. As a result, the PCDm, now in a cantilever shape, detached from the Si wafer (Figure 1a‐v). After rinsing, the free‐standing PCDm cantilevers were transferred onto an SU‐8‐coated Si wafer (Figure 1a‐vi). The SU‐8 layer serves as an adhesive material, permanently securing the PCDm cantilevers. During the transfer process, some PCDm cantilevers were selectively flipped to create BSU‐PCDm cantilevers, while others were placed onto the SU‐8‐coated Si wafer without flipping to create TSU‐PCDm. Due to differences in surface roughness, TSU‐PCDm cantilevers (Figure 1b‐ii) appear rough and dark, whereas BSU‐PCDm cantilevers (Figure 1b‐i) appear smooth and lighter in color (Figure 1a‐vii). Detailed SEM images and microscopic images are provided in Figure S1 and Figure 1b, respectively. Tiny holes were incorporated into the design to facilitate HF penetration and also serve as markers under the AFM camera. The long beam had a width of 300 µm, while the short beam had a width of 150 µm.

### XRD, Morphology, and Topographical Characterization

The structural properties of the PCDm cantilevers were characterized using a PANalytical X'Pert PRO X‐ray diffractometer with Cu Kα radiation in *θ*–2*θ* mode. The X‐ray beam was focused on the stub of the cantilevers, which remained attached to the Si substrate, to maximize the illuminated area of the PCDm. The morphologies of the PCDm cantilevers were examined using a field‐emission scanning electron microscope (FE‐SEM, Carl Zeiss AURIGA). Topographic images were acquired via atomic force microscopy (AFM) (MFP‐3D BioInfinity). The AFM images for both BSU‐ and TSU ‐PCDm were acquired in contact mode with a scan size of 10 µm × 10 µm and a scan rate of 0.1 Hz. Diamond‐coated probes (AIO‐DD, BudgetSensors) were used for the measurements. Among the four cantilevers on each probe, the one with a length of 210 µm and a spring constant of 6.5 N m^−1^ was selected for the analysis. All images were captured at room temperature.

### Bandgap Characterization

X‐ray photoelectron spectroscopy (XPS) was performed using a Kratos Axis Ultra system with a monochromatic Al Kα source (*hv* = 1486.6 eV) and a spot size of 0.7 × 0.4 mm. Absorption spectra were collected using a Cary 7000 universal measurement spectrophotometer with a light spot size of 5 × 1.5 mm. The PCDm used for bandgap characterization were obtained from the same PCDm used to fabricate the PCDm cantilevers. To prepare the PCDm, the PCD thin film grown on a Si/SiO₂ substrate was directly immersed in an HF solution to remove the SiO₂ layer. The detached free‐standing PCDm were then rinsed and dried. For XPS measurements, irregularly shaped PCDm (larger than the XPS spot size) were positioned on a quartz substrate with SU‐8, ensuring either the bottom or top surface facing up. For spectrophotometer measurements, a larger PCDm (10 mm × 6 mm in size) was partially attached to a foreign Si wafer, leaving most of the membrane suspended. During measurement, the sample was placed in a holder with either the bottom or top surface facing the light source, and the light spot was focused on the suspended section of the PCDm to minimize substrate influence. Transmittance (*T*) and reflectance (*R*) were collected from UV–vis measurements, and absorbance (*A*) was calculated using Equation 1: *A* = 1 – *T* – *R*. The absorption coefficient 𝛼 was then determined using the Beer–Lambert law: Equation 2:

(5) $$\alpha =\left(2.302\times A\right)/d$$

where *d* is the film thickness.^[^ ^75^ ^]^

### Chemical and Electrical Characterization

Raman spectra were collected using a Renishaw InVia system, with different settings applied to capture specific peaks. A 514 nm excitation laser wavelength and a 1200 l/mm grating were used to obtain the boron peak, while a 488 nm excitation laser wavelength and a 2400 l/mm grating were employed to achieve higher resolution for the D and G bands. Electrical property measurements of the PCDm were performed using a Keithley 4200‐SCS semiconductor parameter analyzer. A set of TLM patterns were deposited on PCDm, and the current–voltage characteristics were measured under a voltage bias ranging from –0.5 to 0.5 V.

## Conflict of Interest

The authors declare no conflict of interest.

## Supporting information



Supporting Information

## Data Availability

The data that support the findings of this study are available from the corresponding author upon reasonable request.

## References

[1] W. Liu , M. Shamsa , I. Calizo , A. Balandin , V. Ralchenko , A. Popovich , A. Saveliev , Appl. Phys. Lett. 2006, 89, 171915.

[2] R. Kalish , J. Phys. D Appl. Phys. 2007, 40, 6467.

[3] O. Auciello , A. V. Sumant , Diam. Relat. Mater. 2010, 19, 699.

[4] J. J. Alcantar‐Peña , E. de Obaldia , P. Tirado , M. J. Arellano‐Jimenez , J. E. Ortega Aguilar , J. F. Veyan , M. J. Yacaman , Y. Koudriavtsev , O. Auciello , Diam. Relat. Mater. 2019, 91, 261.

[5] I. Krainsky , V. Asnin , Appl. Phys. Lett. 1998, 72, 2574.

[6] S. Łoś , K. Fabisiak , K. Paprocki , M. Szybowicz , A. Dychalska , Sensors 2021, 21, 6113. 34577318 10.3390/s21186113 PMC8473318

[7] F. Zhao , Y. He , B. Huang , T. Zhang , H. Zhu , Materials 2024, 17, 17143437.

[8] V. Sedov , J. Wei , V. Ralchenko , in Novel Aspects of Diamond II: Science and Technology (Eds.: S. Mandal , N. Yang ), Springer Nature, Switzerland, Cham, 2024, 101.

[9] O. Auciello , D. M. Aslam , J. Mater. Sci. 2021, 56, 7171.

[10] V. P. Astakhov , A. Stanley , in Traditional Machining Processes: Research Advances (Ed.: J. P. Davim ), Springer Berlin Heidelberg, Berlin, Heidelberg, 2015, 1.

[11] G. Li , M. Z. Rahim , W. Pan , C. Wen , S. Ding , J. Manuf. Process. 2020, 56, 400.

[12] M. Y. Chernykh , A. A. Andreev , I. S. Ezubchenko , I. A. Chernykh , I. O. Mayboroda , E. M. Kolobkova , Y. V. Khrapovitskaya , J. V. Grishchenko , P. A. Perminov , V. S. Sedov , A. K. Martyanov , A. S. Altakhov , M. S. Komlenok , V. P. Pashinin , A. G. Sinogeykin , V. I. Konov , M. L. Zanaveskin , Appl. Mater. Today 2022, 26, 101338.

[13] Q. Yang , J. Zhao , Y. Huang , X. Zhu , W. Fu , C. Li , J. Miao , Appl. Therm. Eng. 2019, 158, 113804.

[14] S. Hiza , M. Fujikawa , Y. Takiguchi , K. Nishimura , E. Yagyu , T. Matsumae , Y. Kurashima , H. Takagi , M. Yamamuka , in *Proceed. 2019 Int. Conf. Solid State Devices and Mater*. 2019, Aichi, Japan, 2.

[15] Y. Zhou , R. Ramaneti , J. Anaya , S. Korneychuk , J. Derluyn , H. Sun , J. Pomeroy , J. Verbeeck , K. Haenen , M. Kuball , Appl. Phys. Lett. 2017, 111, 041901.

[16] P. Kulha , A. Kromka , O. Babchenko , M. Vanecek , M. Husak , O. A. Williams , K. Haenen , Vacuum 2009, 84, 53.

[17] A. Yamamoto , T. Tsutsumoto , Diam. Relat. Mater. 2004, 13, 863.

[18] A. Balducci , A. D'Amico , C. Di Natale , M. Marinelli , E. Milani , M. E. Morgada , G. Pucella , G. Rodriguez , A. Tucciarone , G. Verona‐Rinati , Sens. Actuators, B 2005, 111–112, 102.

[19] N. Sepulveda , J. Lu , D. M. Aslam , J. P. Sullivan , J. Microelectromech. Syst. 2008, 17, 473.

[20] H.‐Y. Chan , D. M. Aslam , J. A. Wiler , B. Casey , J. Microelectromech. Syst. 2009, 18, 511.

[21] 10.31438/trf.hh2018.67. Y. Guo, C. A. Rusinek, R. Rechenberg, B. Fan, M. F. Becker, W. Li, Solid‐State Sens. Actuators Microsyst. Workshop 2018, 234, 234.

[22] C. Xiao , F.‐C. Hsia , A. Sutton‐Cook , B. Weber , S. Franklin , Carbon N. Y. 2022, 196, 29.

[23] T. Gray , X. Zhang , A. Biswas , T. Terlier , E. F. Oliveira , A. B. Puthirath , C. Li , T. S. Pieshkov , E. J. Garratt , M. R. Neupane , B. B. Pate , A. G. Birdwell , T. G. Ivanov , R. Vajtai , P. M. Ajayan , Carbon N. Y. 2024, 228, 119366.

[24] M. Kim , J.‐H. Seo , U. Singisetti , Z. Ma , J. Mater. Chem. 2017, 5, 8338.

[25] J.‐H. Seo , E. Swinnich , Y.‐Y. Zhang , M. Kim , Mater. Res. Lett. 2020, 8, 123.

[26] Transient Characteristics of β‐Ga2O3 Nanomembrane Schottky Barrier Diodes on Various Substrates , **n.d**.

[27] Influences of Native Oxide on the Properties of Ultrathin Al2O3‐Interfaced Si/GaAs Heterojunctions, **n.d**.

[28] D. Liu , S. J. Cho , J.‐H. Seo , K. Kim , M. Kim , J. Shi , X. Yin , W. Choi , C. Zhang , J. Kim , M. A. Baboli , J. Park , J. Bong , I.‐K. Lee , J. Gong , S. Mikael , J. H. Ryu , P. K. Mohseni , X. Li , S. Gong , X. Wang , Z. Ma , arXiv [physics.app‐ph] 2018.

[29] D. Liu , S. J. Cho , J. Park , J. Gong , J.‐H. Seo , R. Dalmau , D. Zhao , K. Kim , M. Kim , A. R. K. Kalapala , J. D. Albrecht , W. Zhou , B. Moody , Z. Ma , Appl. Phys. Lett. 2018, 113, 011111.

[30] High‐Performance Solar Blind UV Photodetectors Based on Single‐Crystal Si/β‐Ga2O3 p‐n Heterojunction , **n.d**.

[31] J. Lai , M. N. Hasan , E. Swinnich , Z. Tang , S.‐H. Shin , M. Kim , P. Zhang , J.‐H. Seo , J. Mater. Chem. 2020, 8, 14732.

[32] S. D. Janssens , S. Drijkoningen , K. Haenen , Appl. Phys. Lett. 2014, 104, 073107.

[33] S. Salvatori , S. Pettinato , A. Piccardi , V. Sedov , A. Voronin , V. Ralchenko , Materials (Basel) 2020, 13, 3697. 32825659 10.3390/ma13173697 PMC7504279

[34] J. Jing , F. Sun , Z. Wang , L. Ma , Y. Luo , Z. Du , T. Zhang , Y. Wang , F. Xu , T. Zhang , C. Chen , X. Ma , Y. He , Y. Zhu , H. Sun , X. Wang , Y. Zhou , J. K. H. Tsoi , J. Wrachtrup , N. Wong , C. Li , D.‐K. Ki , Q. Wang , K. H. Li , Y. Lin , Z. Chu , Nature 2024, 636, 627. 39695210 10.1038/s41586-024-08218-x

[35] Y. Zheng , M. Muehle , J. Lai , J. D. Albrecht , J.‐H. Seo , J. Vac. Sci. Technol. B Nanotechnol. Microelectron. 2022, 40, 26.

[36] M. N. Hasan , J. Lai , E. Swinnich , Y. Zheng , B. S. Baboukani , P. C. Nalam , J.‐H. Seo , Adv. Electron. Mater. 2021, 7, 2000763.

[37] Y. Zheng , Z. Feng , A. F. M. A. U. Bhuiyan , L. Meng , S. Dhole , Q. Jia , H. Zhao , J.‐H. Seo , J. Mater. Chem. C Mater. Opt. Electron. Devices 2021, 9, 6180.

[38] Y. Zheng , E. Swinnich , J.‐H. Seo , ECS J. Solid State Sci. Technol. 2020, 9, 055007.

[39] A. Gupta , D. Paramanik , S. Varma , C. Jacob , Bull. Mater. Sci. (India) 2004, 27, 445.

[40] D. Das , R. N. Singh , S. Chattopadhyay , K. H. Chen , J. Mater. Res. 2006, 21, 2379.

[41] B. M. Moshtaghioun , F. L. Cumbrera , D. Gómez‐García , J. I. Peña , Sci. Rep. 2019, 9, 13340. 31527636 10.1038/s41598-019-49985-2 PMC6746857

[42] R. W. Armstrong , Metall. Mater. Trans. B 1970, 1, 1169.

[43] M. Mohr , F. Picollo , A. Battiato , E. Bernardi , J. Forneris , A. Tengattini , E. Enrico , L. Boarino , F. Bosia , H.‐J. Fecht , P. Olivero , Diam. Relat. Mater. 2016, 63, 75.

[44] M. L. B. Palacio , B. Bhushan , Mater. Charact. 2013, 78, 1.

[45] C. A. Klein , G. F. Cardinale , Diam. Relat. Mater. 1993, 2, 918.

[46] S. P. Baker , W. D. Nix , J. Mater. Res. 1994, 9, 3131.

[47] A. S. Hall , F. E. Archer , R. I. Gilbert , Engineering Statics, UNSW Press, USA 1999.

[48] R. W. Armstrong , Metallurgical Transactions 1970, 1, 1169.

[49] M. Wiora , K. Brühne , A. Flöter , P. Gluche , T. M. Willey , S. O. Kucheyev , A. W. Van Buuren , A. V. Hamza , J. Biener , H.‐J. Fecht , Diam. Relat. Mater. 2009, 18, 927.

[50] M. A. Hopcroft , W. D. Nix , T. W. Kenny , J. Microelectromech. Syst. 2010, 19, 229.

[51] C. Serre , A. PÃ©rez‐RodrÃ­guez , A. Romano‐RodrÃ­guez , J. R. Morante , J. Esteve , M. C. Acero , J. Micromech. Microeng. 1999, 9, 190.

[52] H. Qin , X. Luan , C. Feng , D. Yang , G. Zhang , Materials 2017, 10, 10121419. 10.3390/ma10121419 PMC5744354 29231902

[53] I. Saeki , T. Ohno , D. Seto , O. Sakai , Y. Sugiyama , T. Sato , A. Yamauchi , K. Kurokawa , M. Takeda , T. Onishi , Mater. High Temp. 2011, 28, 264.

[54] I. Yonenaga , T. Shima , M. H. F. Sluiter , Jpn. J. Appl. Phys. 2002, 41, 4620.

[55] T. Csanádi , M. Vojtko , J. Dusza , Int. J. Refract. Metals Hard Mater. 2020, 87, 105163.

[56] R. J. K. Wood , S. Herd , M. R. Thakare , Tribol. Int. 2018, 119, 491.

[57] A. Nie , Y. Bu , P. Li , Y. Zhang , T. Jin , J. Liu , Z. Su , Y. Wang , J. He , Z. Liu , H. Wang , Y. Tian , W. Yang , Nat. Commun. 2019, 10, 5533. 31797924 10.1038/s41467-019-13378-w PMC6892892

[58] R. S. Sussmann , J. R. Brandon , G. A. Scarsbrook , C. G. Sweeney , T. J. Valentine , A. J. Whitehead , C. J. H. Wort , Diam. Relat. Mater. 1994, 3, 303.

[59] K. Paprocki , A. Dittmar‐Wituski , M. Trzciński , M. Szybowicz , K. Fabisiak , A. Dychalska , Opt. Mater. 2019, 95, 109251.

[60] C. Casiraghi , A. C. Ferrari , J. Robertson , Phys. Rev. B Condens. Matter 2005, 72, 085401.

[61] K. M. McNamara , K. K. Gleason , D. J. Vestyck , J. E. Butler , Diam. Relat. Mater. 1992, 1, 1145.

[62] D. Ballutaud , F. Jomard , T. Kociniewski , E. Rzepka , H. Girard , S. Saada , Diam. Relat. Mater. 2008, 17, 451.

[63] S. Prawer , R. J. Nemanich , Philos. Trans. A Math. Phys. Eng. Sci. 2004, 362, 2537. 15482990 10.1098/rsta.2004.1451

[64] G. Cicala , V. Magaletti , G. S. Senesi , G. Carbone , D. Altamura , C. Giannini , R. Bartali , Mater. Chem. Phys. 2014, 144, 505.

[65] M. T. Nichols , W. Li , D. Pei , G. A. Antonelli , Q. Lin , S. Banna , Y. Nishi , J. L. Shohet , J. Appl. Phys. 2014, 115, 094105.

[66] M. Rycewicz , A. Nosek , D. H. Shin , M. Ficek , J. G. Buijnsters , R. Bogdanowicz , Diam. Relat. Mater. 2022, 128, 109225.

[67] R. Locher , J. Wagner , F. Fuchs , M. Maier , P. Gonon , P. Koidl , Diam. Relat. Mater. 1995, 4, 678.

[68] Y. Sun , X.‐Y. Huang , H. Wang , J. Mater. Eng. Perform. 2016, 25, 1570.

[69] M. Chhowalla , J. Robertson , C. W. Chen , S. R. P. Silva , C. A. Davis , G. A. J. Amaratunga , W. I. Milne , J. Appl. Phys. 1997, 81, 139.

[70] Y. Lu , S. Wang , G. Huang , L. Xi , G. Qin , M. Zhu , H. Chu , J. Mater. Sci. 2022, 57, 3971.

[71] A. C. Ferrari , J. Robertson , Phys. Rev. B Condens. Matter 2000, 61, 14095.

[72] M. Pandey , R. D'Cunha , A. K. Tyagi , J. Alloys Compd. 2002, 333, 260.

[73] J. Wagner , M. Ramsteiner , C. Wild , P. Koidl , Phys. Rev. B Condens. Matter 1989, 40, 1817. 9992043 10.1103/physrevb.40.1817

[74] M. Belousov , V. Krivchenko , P. Minakov , A. Pal , A. Rakhimov , N. Suetin , V. Sen’ , ECS Trans. 2009, 25, 257.

[75] D. F. Swinehart , J. Chem. Educ. 2009, 25, 257.

