# Supplementary material for: Mechanical and Electrical Properties of Free‐standing Polycrystal Diamond Membranes
Source: Adv Sci (Weinh). 2025 Jun 28;12(32):e03986. doi: 10.1002/advs.202503986 (PMC12407314; doi:10.1002/advs.202503986)
Supplement: Supplementary file 1 — Supporting Information [file ADVS-12-e03986-s001.pdf]

ADVANCED SCIENCE Open Access

Supporting Information

for *Adv. Sci.*, DOI 10.1002/advs.202503986

Mechanical and Electrical Properties of Free-standing Polycrystal Diamond Membranes

*Chenyu Wang, Dmitry Shinyavskiy, Luke Suter, Zubaida Altikriti, Quanxi Jia, Matthias Muehle and Jung-Hun Seo**

Supporting Information

for

Mechanical and Electrical Properties of Free-standing Polycrystal Diamond Membranes

Chenyu Wang[1], Dmitry Shinyavskiy[1], Luke Suter[2], Zubaida Altikriti[1], Quanxi Jia[1], Matthias Muehle[2], Jung-Hun Seo[1,*]

[1]Department of Materials Design and Innovation, University at Buffalo, The State University of New York, Buffalo, NY 14260
[2]Fraunhofer USA Inc., Center Midwest, East Lansing, MI 48824, USA

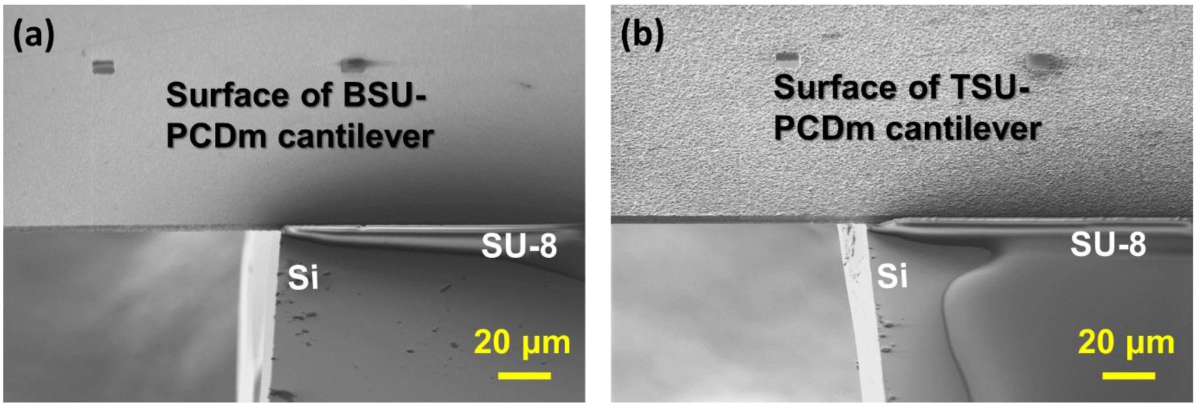

Figure S1. SEM images of **(a)** the BSU-PCDm cantilever, and **(b)** the TSU-PCDm cantilever attached to the SU-8 coated Si substrate.

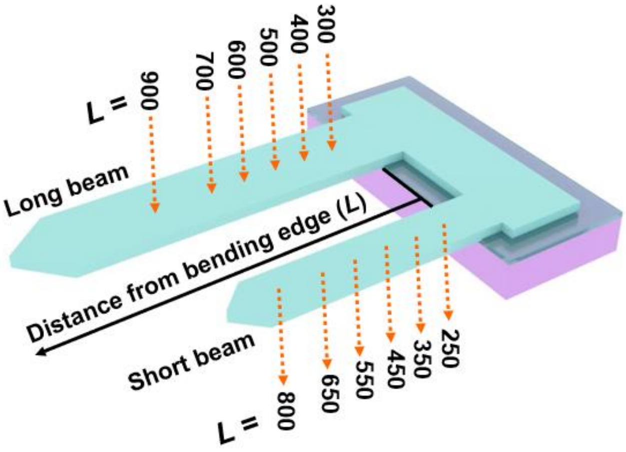

Figure S2. A scheme of PCDm cantilevers for their mechanical property characterization.

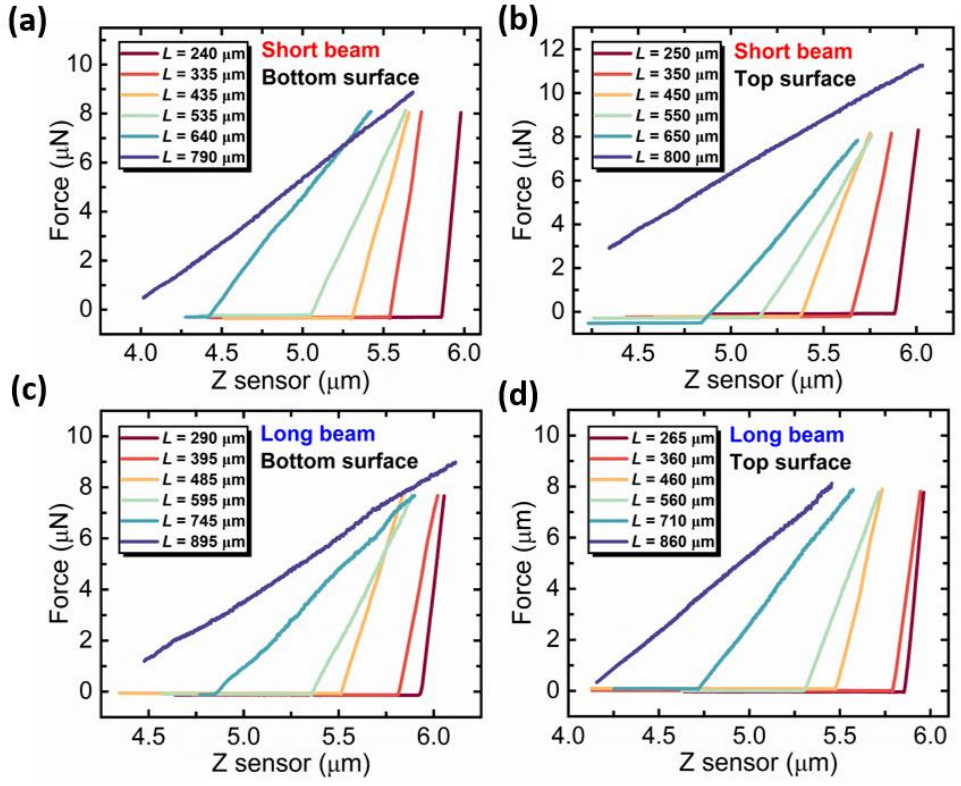

Figure S3. Force plots of PCDm cantilevers. **(a)** The short BSU-PCDm cantilever, **(b)** the short TSU-PCDm cantilever, **(c)** the long BSU-PCDm cantilever, and **(d)** the long TSU-PCDm cantilever.

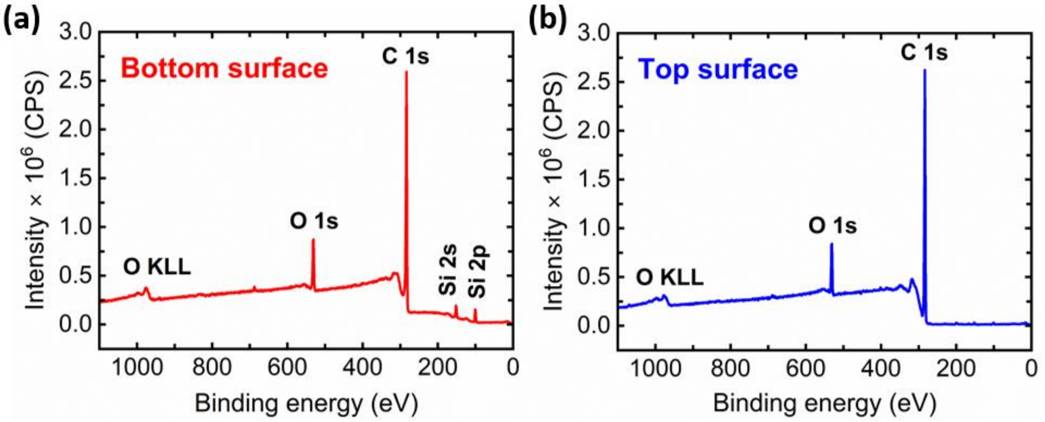

Figure S4. XPS spectrum measured from the surface of **(a)** BSU- and **(b)** TSU- PCDm.

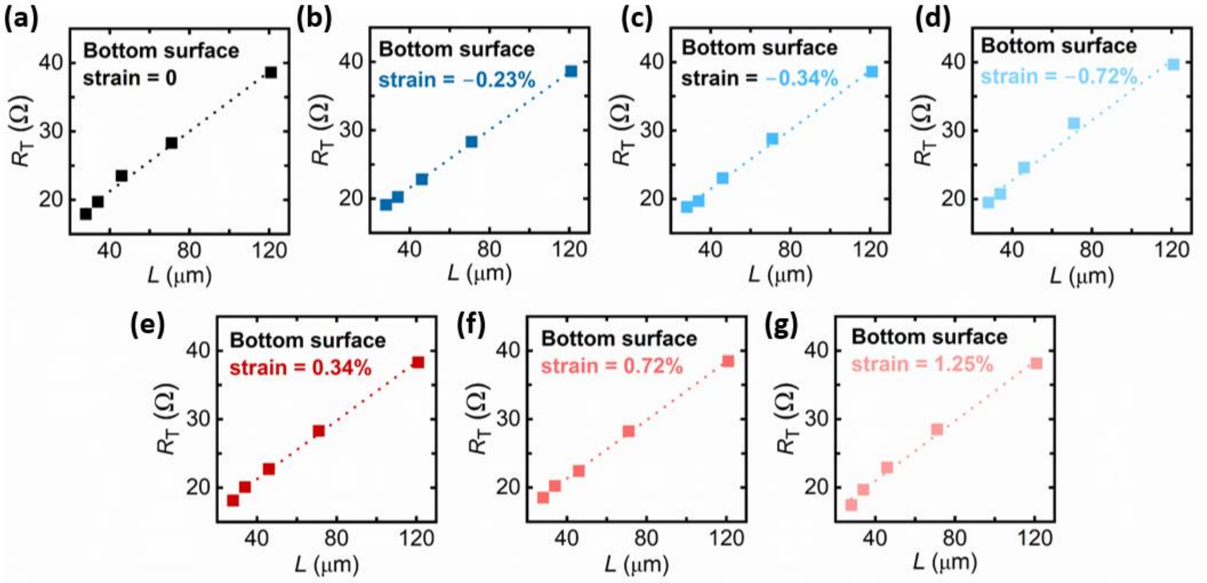

Figure S5. TLM plots for the BSU- PCDm under different strain conditions. **(a)** strain = 0%. **(b)** strain = –0.23%. **(c)** strain = –0.34%. **(d)** strain = –0.72%. **(e)** strain = 0.34%. **(f)** strain = 0.72%. **(g)** strain = 1.25%.

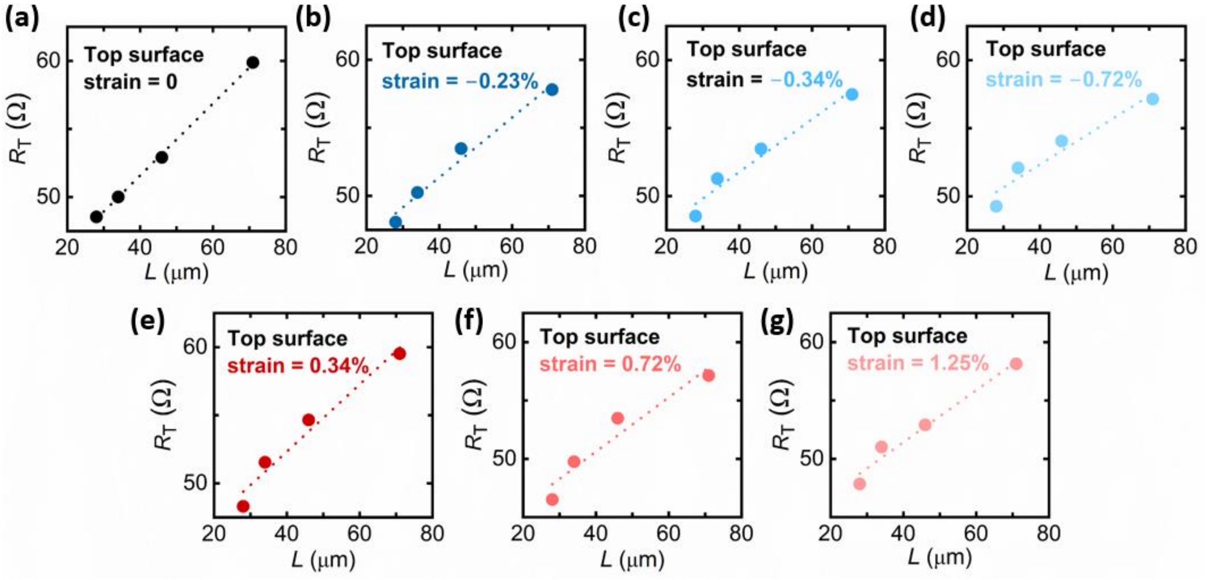

Figure S6. TLM plots for TSU- PCDm under different strain conditions. **(a)** strain = 0. **(b)** strain = –0.23%. **(c)** strain = –0.34%. **(d)** strain = –0.72%. **(e)** strain = 0.34%. **(f)** strain = 0.72%. **(g)** strain = 1.25%.

Table S1. Parameters of the diamond-coated AFM tip.

**Length (μm)**	**Frequency (kHz)**	**Spring constant (N/m)**
100	450	100

Table S2. sp^2 and CH_x fractions of BSU- and TSU- PCDm cantilevers calculated from XPS and Raman spectra.

**sp^2 / sp^3**	**Bottom**	**Top**	**CH_x fraction**	**Bottom**	**Top**
**XPS**	0.104	0.059	**XPS (CH_x / sp^3)**	0.263	0.237
**Raman**	0.008	0.004	**Raman (PL slope) (a.u./cm^{-1})**	19.99	16.58
